# Expression of AIM2 in Rheumatoid Arthritis and Its Role on Fibroblast-Like Synoviocytes

**DOI:** 10.1155/2020/1693730

**Published:** 2020-10-26

**Authors:** Yong Chen, Qiu Fujuan, Ensheng Chen, Beijia Yu, Fangfang Zuo, Yi Yuan, Xiaofeng Zhao, Changhong Xiao

**Affiliations:** Rheumatology Department, Integrated Traditional Chinese and Western Medicine Hospital, Southern Medical University, Guangzhou 510315, China

## Abstract

**Objectives:**

To determine differences in AIM2 inflammasome expression levels between rheumatoid arthritis (RA) and osteoarthritis (OA) and to investigate the role of AIM2 in RA fibroblast-like synoviocytes (RA-FLS).

**Methods:**

Serum AIM2 levels among health controls (HC, *n* = 20), OA (*n* = 25), and RA (*n* =49) patients were compared via ELISA. The different expression levels of AIM2, ASC, caspase-1, and IL-1*β* between RA and OA synovium were semiquantified by qRT-PCR and immunohistochemical (IHC) staining. IHC staining was recorded by *H* scores, and its correlation with the ESR and CRP levels of RA patients was determined. SiRNA AIM2 was transferred to RA-FLS and its effects on the proliferation and migration via CCK-8 assay and Transwell test, respectively.

**Results:**

In RA sera, the HC expressed higher level of AIM2 than OA and RA patients, and ASC, caspase-1, and IL-1*β* expressed higher in RA patients than HC; no significant differences were observed between sera of OA and RA patients. However, in affected knee synovium, AIM2, ASC, caspase-1, and IL-1*β* were expressed higher in RA than that of OA. Moreover, the *H* scores of AIM2, ASC, and IL-1*β* were positively correlated with the ESR and CRP levels in RA patients. The proliferation of FLS was significantly inhibited after transferring with AIM2 siRNA to FLS. There were no differences in apoptosis and migration assay between the si-AIM2 group and the control group.

**Conclusion:**

AIM2 inflammasome pathway involves in the pathogenesis of RA. si-AIM2 inhibits the proliferation of RA-FLS, which may be a promising therapeutic strategy for the treatment of RA.

## 1. Introduction

Rheumatoid arthritis (RA) is a chronic autoimmune inflammatory disease characterised by the hyperproliferation of synovial cells, infiltration of mononuclear cells into the synovium, and early destruction of the articular cartilage and bone, causing damage to the musculoskeletal system and consequently loss of physical ability and quality of life [1, 2]. Past studies regarding the genetic architecture of RA have been well characterised through conventional and genome-wide approaches, and more than 100 loci were found to be associated with disease risk and progression [3], among which, absent in melanoma 2 (AIM2) was one of the genes detected [4, 5].

AIM2 is involved in innate immune responses through the recognition of cytosolic double-stranded DNA and the induction of caspase-1-activating inflammasome formation in macrophages [6]. Upon binding to DNA, AIM2 is thought to undergo oligomerization and to associate with PYCARD (PYD and CARD domain containing/ASC, apoptosis-associated speck-like protein containing a CARD) initiating the recruitment of caspase-1 precursors and the processing of interleukin- (IL-) 1*β* and IL-18 [7, 8]. Inappropriate recognition of cytoplasmic self-DNA by AIM2 contributes to the development of psoriasis, dermatitis, arthritis, and other autoimmune or inflammatory diseases [9]. AIM2 was reported upregulated in RA synovium than that of OA [5], and AIM2 inflammasome plays role in the activation of neutrophils [10] and also vascular dysfunction [11]. However, studies regarding AIM2 inflammasome on arthritis were still limited, especially investigations on clinical patients have not been reported, which leads to the AIM2 inflammasome in the pathogenesis of RA is not well demonstrated.

In this study, we investigate the differential expression partial of the AIM2 pathway associated proteins, including AIM2, ASC, caspase-1, and IL-1*β*, from their features in plasma and synovium of OA and RA from mRNA and protein aspects. RA-fibroblast-like synoviocytes (RA-FLS) is critical to the pathogenesis of RA as it may develop a uniquely aggressive phenotype that increases invasiveness into the extracellular matrix and further exacerbates joint damage [12]. Therefore, in this study, we also attempt to observe si-AIM2's role in the viability of RA-FLS.

## 2. Materials and Methods

### 2.1. Patients' Serum for Enzyme Linked Immunosorbent Assay (ELISA)

We enrolled 49 patients diagnosed with RA [13] and 25 patients diagnosed with OA [14] with comprehensive medical records from the Rheumatology Department in the Integrated Traditional Chinese and Western Medicine Hospital, Southern Medical University, China, between October 2017 and December 2018. Exclusion criteria included other autoimmune diseases, acute inflammation, fever, thyroid disease, diabetes, and severe liver and kidney diseases. Twenty health volunteers were enrolled as health controls (HC). Patients' sera were collected for ELISA assays of AIM2, ASC, caspase-1, and IL-1*β* (R&D Systems, USA). The ELx808TM absorbance microplate reader was used to measure the absorbance values at 450 nm. Concentrations of proteins in the samples were calculated using the standard curve for each protein. The general information of the patients recruited for this study is given in Table 1. All subjects provided written informed consent for participation in the study as approved by the ethical committee of each institutional review board.

### 2.2. Patients' Synovium for Immunohistochemical (IHC) Staining

Arthroscopic surgery was performed on 41 RA and 26 OA patients for therapeutic purposes, and the patients' general information is shown in Table 2. Regular streptavidin biotin-based immunoperoxidase staining for AIM2, ASC, caspase-1, and IL-1*β* was performed to formalin fixed, paraffin-embedded pathology keen synovium specimens. The *H* score was then applied to quantify the staining intensity [15].

RA: rheumatoid arthritis; OA: osteoarthritis; ESR: erythrocyte sedimentation rate; CRP: C-reactive protein. ^∗∗^*p* < 0.01, ^∗∗∗^*p* < 0.001.

### 2.3. Patients' Synovium for Quantitative Real-Time Polymerase Chain Reaction (qPCR)

Synovium specimens taken from 10 RA patients and 9 OA patients were obtained via knee arthroscopy, and the patients' general information is given in Table 3. Specimens were soaked in TRIzol® Reagent (Thermo Scientific, USA) after removing adipose tissue in an aseptic environment and were then stored in a -20°C refrigerator in preparation for qPCR of the mRNA AIM2, ASC, caspase-1, and IL-1*β*. qPCR was then done to evaluate the relative expression of the mRNAs of AIM2, ASC, caspase-1, and IL-1*β* in FLS after transferring AIM2 siRNA. The primers used in the amplification of the target mRNAs are listed in Table 4.

RA: rheumatoid arthritis; OA: osteoarthritis; ESR: erythrocyte sedimentation rate; CRP: C-reactive protein. ^∗^*p* < 0.05, ^∗∗^*p* < 0.01.

### 2.4. Immunofluorescent Staining for AIM2

In regard to immunofluorescent staining, both groups of RA and OA patients were blocked with normal goat serum in 0.01 M phosphate-buffered saline for 1 hour. The primary rabbit anti-rat AIM2 antibody (1 : 200) was incubated overnight at 4°C together with mouse antihuman vimentin (1 : 400). The following day, the sections were incubated for 60 minutes at 37°C with FITC-conjugated goat and anti-mouse antibodies (1 : 1000) as well as anti-rabbit antibodies (1 : 1000). The nuclei of cells were stained with DAPI. Observations were done under fluorescence microscopy for which the corresponding results were recorded.

### 2.5. AIM2 siRNA Preparation and Transfection

AIM2 siRNA were produced by the Ribobio Company, China. RA-FLS at 50–70% confluency were transfected with siRNAs (20 nM) as previously described [16]. The following siRNA sequences were used: untargeted control siRNA (5′-GCGCUAUCCAGCUUACGUAUU-3′) and AIM2 siRNA (5′-GAGCTCTTCACCACTTTCA-3′).

### 2.6. Isolation and Culture of Fibroblast-Like Synoviocytes (FLS)

FLS were derived from synovial tissue specimens that were harvested from patients using microarthroscopy. They were then isolated using enzyme digestion, which was subsequently cultured in Dulbecco's Modified Essential Medium (DMEM) containing 10% fetal bovine serum (FBS, Invitrogen) and antibiotics (penicillin and streptomycin) at 37°C with 5% CO_2_. The cells cultured between passages 4 and 9 were used in this study. The cells were frozen with a cell-freezing medium and stored in a -80°C freezer until they were needed.

### 2.7. Cell Counting Kit-8 (CCK-8) Assay

RA-FLS were seeded in 96-well plates (1 × 10^4^ cells/well) and treated with siRNA for 24, 48, and 72 hours, respectively. Cell viability was determined using Cell Counting Kit-8 (CCK-8, Dojindo Molecular Technology, Japan) according to the manufacturer's protocol. Finally, the optical density was monitored at 450 nm by a Multiskan Spectrum Microplate Reader (Thermo, USA).

### 2.8. Flow Cytometry in the Evaluation of Apoptosis

Flow cytometry was performed to evaluate the effects of siRNA AIM2 on FLS apoptosis. Accordingly, 48 hours after FLS was treated by siRNA AIM2, of 6-well plates in both NC and siRNA AIM2 group; the cells were collected (approximately 3 × 10^5^/well), washed twice with PBS, and resuspended in a 500 *μ*l 1 × binding buffer. They were then mixed with 5 *μ*l of Annexin-V-fluorescein isothiocyanate (FITC) and 5 *μ*l of propidium iodide (PI) and were detected by a flow cytometer (BD LSRFortessa™, USA). The scatter diagram demonstrated a distribution as follows: Q3: healthy cells (FITC-/PI-), Q2: apoptotic cells at an advanced stage (FITC+/PI+), and Q4: apoptotic cells at an early stage (FITC+/PI-). The apoptosis rate is equal to the ratio of apoptotic cells to the total cells in Q4 plus the ratio of apoptotic cells to the total cells in Q2. Each experiment was conducted three times [17].

### 2.9. Transwell Test

For the Matrigel invasion assay, cells at the logarithmic growth phase were digested, collected, resuspended, and diluted into a concentration of 3 × 10^4^/ml in a serum-free medium. The cell suspension (200 *μ*l) was added to the upper chamber and coated with Matrigel (BD Bioscience) diluted with DMEM media (1 : 3), while 500 *μ*l DMEM media containing 10% FBS was added to the lower chamber. Following incubation for 12 h at 37°C, the cells in the upper membrane were discarded, and cells on the lower membrane were fixed using 4% Paraformaldehyde for 25 min and stained with 0.1% crystal violet (Beyotime, USA) for 10 min. Next, migrated RA-FLS five random fields were counted.

### 2.10. Automated Electrophoresis Western Blot Analysis

FLS were seeded at 1 − 2 × 10^5^ cells per well in 6-well plates and incubated for adherence. The medium in the wells was replaced with fresh medium containing metformin (5 mM) or saline for 48 h. After aspiration of the medium, the cell monolayers were rinsed with 1 ml ice-cold PBS and lysed in 80 *μ*l of lysis buffer (20 mM Tris–HCl pH 7.5, 150 mM NaCl, 1 mM EDTA, 1 mM EGTA, 1% (*v*/*v*) TritonX100, 2.5 mM sodium pyrophosphate, and 1 mM *β*-glycerophosphate), which were then supplemented with fresh 1 mM Na_3_VO_4_ and 1 mM dithiothreitol containing a 1 X protease inhibitor cocktail (Roche Molecular Biochemicals, Basel, Switzerland). The lysates were precleared by centrifugation at 18000 g for 15 min at 4°C. The supernatants were then collected, and the protein concentrations were measured using a Bradford assay (Thermofisher, Massachusetts, USA). Next, the lysates were adjusted to 5 mg/ml protein concentration. Capillary electrophoresis Western blot analysis was carried out using the manufacturer's reagents provided in the user manual (ProteinSimple WES, San Francisco, USA). Briefly, 5.6 *μ*l of the cell lysate was mixed with 1.4 *μ*l of the fluorescent master mix and heated at 95°C for 5 min. The samples, blocking reagent, wash buffer, antibody of tubulin (1 : 100 R&D Systems) and AIM2 (1 : 50 R&D Systems), secondary antibody, and chemiluminescent substrate were dispensed into the microplates. Then, electrophoretic separation and immunodetection were automatically performed using the default settings. The data was analyzed using the built-in Compass software for SW 4.0. The truncated and full-length AIM2 and tubulin intensities (area under the curve) were normalized to that of the tubulin peak (control). In most of the figures, electropherograms are represented as pseudo-blots, which were generated using the Compass software.

### 2.11. Statistical Analysis

The statistical analysis was conducted using the GraphPad Prism 7.0 software. All data were denoted as mean ± SD. Differences between two groups were evaluated for statistical significance using Student's *t*-test. Kruskal-Wallis and Dunn's multiple comparison post hoc tests were used to evaluate the differences among three or more groups. Correlations were evaluated using Spearman's Rank correlation test, where *p* ≤ 0.05 was considered as statistically significant.

## 3. Results

### 3.1. No Significant Difference of AIM2 Levels in Sara between OA and RA

**Figure 1:**
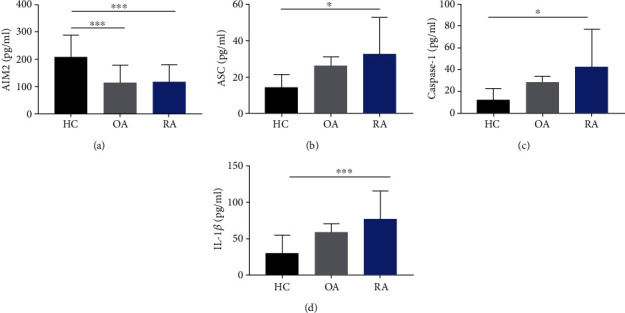
The differences in AIM2 pathway-related molecules in sera among HCs, OA, and RA patients. The HCs expressed obviously higher level of AIM2 than OA and RA patients (a), and ASC, caspase-1, and IL-1*β* expressed obviously higher in RA patients than HCs (b–d). No statistical differences of these molecules between OA and RA (a–d). ^∗^*p* < 0.05, ^∗∗∗^*p* < 0.001, and ^∗∗∗∗^*p* < 0.0001.

Through ELISA, no differences in the AIM2 expression were observed between the sera of OA and RA; however, the HCs expressed obviously higher level of AIM2 than OA and RA patients (Figure 1(a)). The downstream molecules in AIM2 inflammasome pathway including ASC, caspase-1, and IL-1*β* expressed obviously higher in RA patients than HCs. Although there were tendency of increasing expression from OA to RA, no statistical significance was observed (Figures 1(b)–1(d)).

### 3.2. AIM2 Was Expressed More in RA Synovium than in That of OA

**Figure 2:**
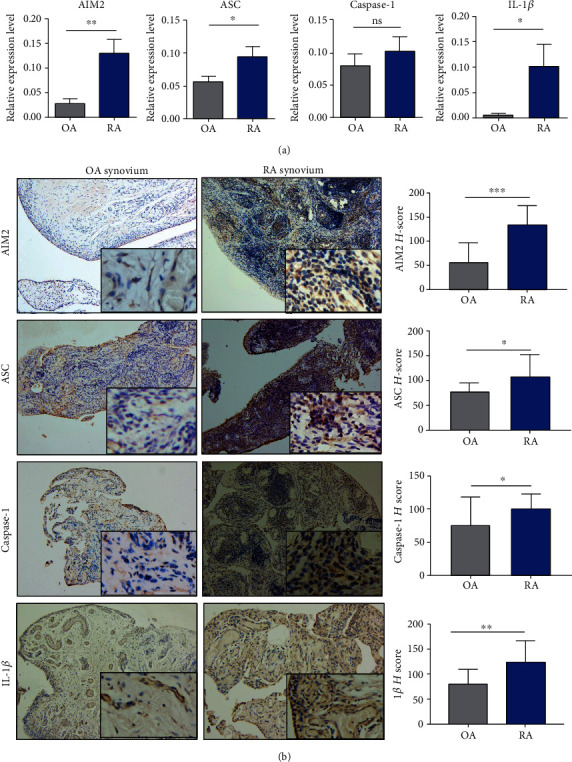
The differences in AIM2 pathway-related molecules in synovium among OA and RA. mRNA AIM2 and AIM2 pathway-related proteins including ASC and IL-1*β* were expressed higher in RA than in OA; caspase-1 was higher in RA than in OA without statistical significance (a). *H* score for the synovium of RA, AIM2, and AIM2 pathway-related proteins including ASC, caspase-1, and IL-1*β* was more expressed than that of OA (b). ^∗^*p* < 0.05, ^∗∗^*p* < 0.01, and ^∗∗∗∗^*p* < 0.0001.

The relative expression levels of mRNA AIM2 as well as the AIM2 pathway-related proteins including ASC and IL-1*β* were higher in the local synovium of RA than that of OA. Moreover, the relative expression of caspase-1 was higher in RA than in OA without statistical significance (Figure 2(a)).

**Figure 3:**
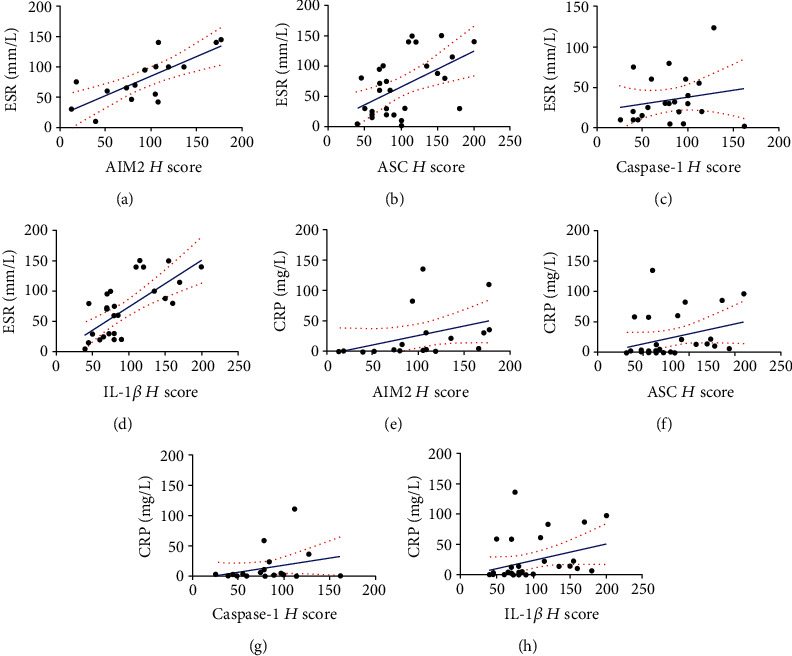
*H* scores of AIM2, ASC, and IL-1*β* were positively correlated with ESR and CRP. AIM2 demonstrated a positive correlation with ESR (*r* = 0.74, *p* = 0.001, 95% CI: 0.38-0.9) and CRP (*r* = 0.65, *p* = 0.003, 95% CI: 0.25-0.86) (a, e). The positive correlations between ASC and ESR (*r* = 0.5, *p* = 0.005, 95% CI: 0.16-0.74) as well as CRP (*r* = 0.42, *p* = 0.02, 95% CI: 0.05-0.69) were detected (b, f). The positive correlations between IL-1*β* and ESR (*r* = 0.62, *p* = 0.0004, 95% CI: 0.31-0.81) and CRP (*r* = 0.41, *p* = 0.02, 95% CI: 0.05-0.67) were detected. The correlations between CASPSE-1 and ESR as well as CRP were not statistically significant (*p* > 0.05) (c, d).

Through the *H* scores in the synovium of RA, AIM2, and the AIM2 pathway-related proteins like ASC, caspase-1, and IL-1*β* were more expressed than that of OA (Figure 2(b)). AIM2, ASC, caspase-1, and IL-1*β* were positively correlated to the clinical features of RA, as indicated by the ESR and CRP levels (Figure 3).

**Figure 4:**
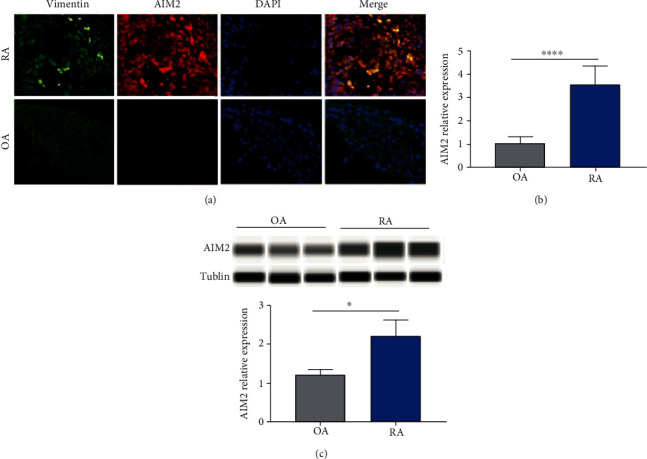
AIM2 is expressed more in RA-FLS than in OA. AIM2 expressed in the cytoplasm of FLS, which is higher in RA-FLS than in OA-FLS ((a) vimentin was labelled as green, AIM2 as red, and nuclei as blue by immunofluorescent staining). The relative expression of AIM2 in mRNA and protein levels was higher in RA-FLS than in OA (b, c). ^∗^*p* < 0.05 and ^∗∗∗∗^*p* < 0.0001.

By performing IHC staining, AIM2 and AIM2 pathway-related proteins were detected in the nucleus and cytoplasm, which are seen in most types of cells including macrophages, lymphocytes, and FLS. As FLS produce cytokines that perpetuate inflammation and proteases that contribute to cartilage destruction, it plays a critical role in the pathogenesis of RA [18]. Hence, this study focuses on the AIM2 pathway in FLS. It was confirmed that AIM2 could be expressed in the cytoplasm of FLS (Figure 4(a)), and AIM2 and its mRNA are relatively higher in RA-FLS than in OA (Figures 4(b) and 4(c)).

### 3.3. AIM2 siRNA Inhibited Proliferation but Not Migration and Apoptosis of RA-FLS

**Figure 5:**
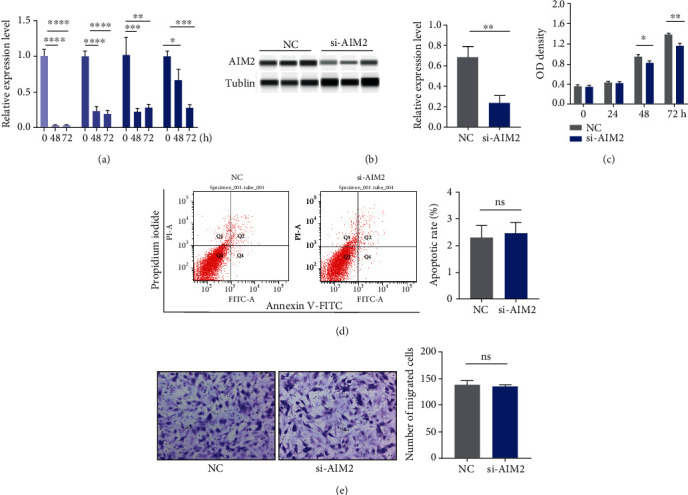
Proliferation, apoptosis, and migration assay of RA-FLS after si-AIM2 transfection. AIM2 and its downstream proteins including ASC, caspase-1, and IL-1*β* were successfully silenced after 24 h of transfection (a). Western blot confirmed the successful silencing of the AIM2 expression by si-AIM2 (b). The proliferation of RA-FLS was inhibited after 48 h of transfection (c). There were no significant differences between the NC and si-AIM2 groups in apoptosis and migration assay (d, e). ^∗^*p* < 0.05, ^∗∗^*p* < 0.01, ^∗∗∗^*p* < 0.001, and ^∗∗∗∗^*p* < 0.0001.

As we designed siRNAs for AIM2, its efficacy demonstrated its successful silencing of the mRNA AIM2 expression along with downstream molecules including ASC, caspase-1, and IL-1*β* (Figure 5(a)). Western blot confirmed the successful silencing of the AIM2 expression by si-AIM2 (Figure 4(b)). By utilizing CCK-8 assay, AIM2 siRNA was found to inhibit the proliferation of RA-FLS (Figure 5(c)). However, no significant difference in the apoptotic rate or number of migrated cells was observed among the normal control and si-AIM2 groups (Figures 5(d) and 5(e)).

## 4. Discussion

As one of the most commonly encountered autoimmune diseases, both innate and adaptive immune responses participate in the pathogenesis of RA [19]. AIM2 has been mostly studied in cells of the immune system or within the context of infection; however, its role in other tissues remains to be investigated. AIM2 acts as an important component of inflammasome that senses potentially dangerous cytoplasmic DNA, leading to the activation of the ASC pyroptosome, caspase-1, and processing of IL-1*β* and IL-18 [20]. A plethora of MtDNA and damaged DNA have been observed in the synovial fluid of RA patients [21]. Although the role of AIM2 has been investigated in the context of DNAse deficiency mices, the role of the AIM2 pathway in the pathogenesis of RA patients has rarely been investigated. Besides the distinct pathological differences, OA patients also have slightly increased inflammatory status and pannus-like tissue in affected synovium [22], which make them qualified to be the controls for study of RA. Therefore, this study compared the AIM2 pathway-associated proteins including AIM2, ASC, caspase-1, and IL-1*β* in the sera and synovium of RA and OA.

Accordingly, it was found that AIM2, ASC, caspase-1, and IL-1*β* were without a significant difference between OA and RA in plasma; however, upregulation was observed in affected joints in the RA synovium than that of OA. Although AIM2 is mostly expressed in the cytoplasm, it may also be detected in serum [23]. We first analyzed the serum levels of AIM2 among HC, OA, and RA patients. However, the plasma levels of AIM2 inflammasome-associated molecules such as ASC, caspase-1, and IL-1*β* among the OA and RA patients have no significant difference, although expressed higher in RA than HC, which is reasonable according to the knowledge that they participates in inflammation process [24]. A meta-analysis reported that a high level of expression of AIM2 in peripheral blood mononuclear cells (PBMC) was observed in RA patients [25]. It is surprisingly to observe the decreased levels of AIM2 in RA than HC in our study. Recently, Mendez-Frausto et al. [26] observed that AIM2 was reduced at a systemic level in patients with RA, and the monocytes of RA patients were found to be prone to releasing IL-1*β* in the absence of AIM2 inflammasome signals. These findings were in accordance with our results of ELISA; however, reasons regarding the decreased levels of AIM2 according to disease activity in serum remain unclear. Next, the mRNA and protein levels of AIM2 and its downstream proteins including ASC, caspase-1, and IL-1*β* in RA were higher in RA than that in OA synovium. The results are consistent with previous investigations [27–29]. However, our data is the first study that investigates the expression of AIM2 inflammasomes in the synovium of inflamed joints from clinical patients. Studies showed that OA and RA pannus have similar qualitative metabolic characteristics and proinflammatory cytokine response; however, OA synovial tissue and pannus had lower production of proteoglycans, type II collagen, and proinflammation cytokines than RA [30, 31]. AIM2 in RA patients' synovium was elevated compared to a less severe form of OA inflammatory. Furthermore, the expression of AIM2 inflammasome-related molecules were observed positively correlated with the ESR and CRP, which confers obvious statistical significance. Thus, we speculate that AIM2 pathway contributes to the inflammatory pathogenesis of RA.

AIM2 plays a different role and acts via different signalling pathways under different circumstances according to studies regarding cancer that have been previously published. The AIM2 pathway serves a putative role in tumorigenic reversion and may control cell proliferation [32, 33]. AIM2 expression suppressed the proliferation and tumorigenicity of human breast cancer cells, and AIM2 gene therapy inhibited mammary tumor growth in an orthotropic tumor model. However, AIM2 promotes cell growth in non-small-cell lung cancer [32]. AIM2 expression greatly suppressed nuclear factor-kappa B transcriptional activity and desensitized tumor necrosis factor-alpha (TNF-*α*) mediated nuclear factor-kappa B activation [34]. Inspired by the role of AIM2 in cancer research, we focused our study on FLS, which mimics the cancer cell biobehaviours of proliferation and invasion and is currently regarded as a crucial effector in the pathogenesis of RA. In IHC staining, we can observe multiple cells including FLS could be stained positively with AIM2 and immunofluorescent staining confirmed the expression of AIM2 in FLS. Then, qPCR and automated electrophoresis Western blot analysis showed RA-FLS express higher level of AIM2 than that of OA. In recent decades of research, the role of FLS in pathogenesis of rheumatoid arthritis has been arising more and more attention and regarded as key effector cells [35]. The synovium in RA transforms from a quiescent relatively acellular structure to a hyperplastic, invasive tissue teeming with immunocompetent cells, to form pannus, which contributes to destructing joint cartilage through their production of inflammatory cytokines [18]. Thus, we focus on whether AIM2 plays a role in viability of RA-FLS. The results demonstrated that the proliferation of FLS was inhibited when AIM2 was silenced in FLS by siRNA. However, the apoptosis and migration were unaffected.

In summary, based on the study of AIM2 pathway, we acknowledged its unregulated change in RA, which is more obvious in local affected joints. Furthermore, we learned AIM2 is upregulated in FLS, and suppress its expression leads to inhibiting effect on FLS proliferation, which suggest AIM2 a promising therapeutic target in RA. The corresponding underlying mechanisms of the results obtained in this study require further investigation.

## Figures and Tables

**Table 1:** General information of patients and health controls recruited for ELISA in this study.

General information	HC (*n* = 20)	OA (*n* = 25)	RA (*n* = 49)
Gender (male/female)	8/12	7/18	11/38
Age (y/o)	45.87 ± 11.35	55.48 ± 9.69	50.87 ± 9.35
Disease course (years)	/	6.41 ± 8.88	7.27 ± 5.11
ESR (mm/h)	/	34.7 ± 24.88	56.12 ± 40.36^∗^
CRP (mg/L)	/	2.21 ± 2.76	12.16 ± 20.36^∗^

HC: health controls; RA: rheumatoid arthritis; OA: osteoarthritis; ESR: erythrocyte sedimentation rate; CRP: C-reactive protein. ^∗^*p* < 0.05.

**Table 2:** General information of patients recruited for IHC in this study.

General information	RA (*n* = 41)	OA (*n* = 26)
Gender (male/female)	6/35	6/20
Age (y/o)	51.60 ± 2.42	60.62 ± 2.30
Disease course (years)	6.54 ± 0.89	6.42 ± 1.43
ESR (mm/h)	79.68 ± 6.90	38.38 ± 6.64^∗∗∗^
CRP (mg/L)	30.26 ± 5.44	7.015 ± 4.22^∗∗^

**Table 3:** General information of patients recruited for RT-qPCR in this study.

General information	RA (*n* = 10)	OA (*n* = 9)
Male to female	2 : 8	5 : 4
Age (years)	54.2 ± 8.65	66.6 ± 8.61^∗∗^
ESR (mm/h)	86.5 ± 50.5	37.8 ± 29.3^∗∗^
CRP (mg/L)	19.1 ± 13.63	5.13 ± 7.19^∗^

**Table 4:** qPCR primers used in this study.

Primer name	Sense primer/sequence	Antisense primer
AIM2	AGCAAGATATTATCGGCACAGTG	GTTCAGCGGGACATTAACCTT
ASC	CCTACTGTTCTTTCTGTGGGAAG	CGAGGTCGTCAGCCATCAC
CASPASE-1	TTTCCGCAAGGTTCGATTTTCA	GGCATCTGCGCTCTACCATC
IL-1*β*	TTCGACACATGGGATAACGAGG	TTTTTGCTGTGAGTCCCGGAG
GAPDH	ACAACTTTGGTATCGTGGAAGG	GCCATCACGCCACAGTTTC

## Data Availability

The data used to support the findings of this study are available from the corresponding author upon request.
